# β-thalassemia intermedia in a Brazilian patient with - 101(C > T) and codon 39 (C > T) mutations

**DOI:** 10.1590/S1516-31802003000100007

**Published:** 2003-01-02

**Authors:** Sylvia Morais de Sousa, Letícia Khater, Luís Antônio Peroni, Karine Miranda, Marcelo Jun Murai, Dulcinéia Martins Albuquerque, Paulo Arruda, Sara Terezinha Ollala Saad, Fernando Ferreira Costa

**Keywords:** Beta thalassemia, Mutations, Beta globin, β-talassemia, Mutações, Globina β

## Abstract

**CONTEXT:**

We verified molecular alterations in a 72-year-old Brazilian male patient with a clinical course of homozygous β-thalassemia intermedia, who had undergone splenectomy and was surviving without regular blood transfusions. The blood cell count revealed microcytic and hypochromic anemia (hemoglobin = 6.5 g/dl, mean cell volume = 74 fl, mean cell hemoglobin = 24 pg) and hemoglobin electrophoresis showed fetal hemoglobin = 1.3%, hemoglobin A = 6.78% and hemoglobin A = 79.4%.

**OBJECTIVE:**

To identify mutations in a patient with the symptoms of β-thalassemia intermedia.

**DESIGN:**

Molecular inquiry into the mutations possibly responsible for the clinical picture described.

**SETTING:**

The structural molecular biology and genetic engineering center of the Universidade Estadual de Campinas, Campinas, Brazil.

**PROCEDURES:**

DNA extraction was performed on the patient's blood samples. The polymerase chain reaction (PCR) was done using five specific primers that amplified exons and the promoter region of the β globin gene. The samples were sequenced and then analyzed via computer programs.

**RESULTS:**

Two mutations that cause the disease were found: -101 (C > T) and codon 39 (C > T).

**CONCLUSIONS:**

This case represents the first description of −101 (C > T) mutation in a Brazilian population and it is associated with a benign clinical course.

## RESUMO

**CONTEXTO:**

Neste trabalho foi estudada a alteração molecular em um paciente brasileiro de 72 anos com talassemia β homozigótica com evolução clínica intermediária, esplenectomizado e necessitando de transfusões ocasionais. Os dados hematológicos mostravam anemia microcítica e hipocrômica (Hemoglobina = 7,9 g/dl, Volume Corpuscular Médio = 76fl, Hemoblobina Corpuscular Média = 26 pg) e a eletroforese de hemoglobina revelou Hemoglobina Fetal = 14,2%, Hemoglobina A_2_ = 6,2% e Hemoglobina A = 79,4%.

**OBJETIVO:**

Identificar as duas mutações envolvendo um paciente com sintomas de talassemia beta intermediária.

**TIPO DE ESTUDO:**

Investigação molecular das possíveis mutações responsáveis pelo quadro clínico descrito.

**LOCAL:**

Centro de Biologia Molecular e Engenharia Genética, Universidade Estadual de Campinas, Campinas, São Paulo, Brasil.

**PROCEDIMENTOS:**

Após a extração de DNA do sangue deste paciente, foi feita reação de polimerase em cadeia (*polymerase chain reaction*, PCR) utilizando cinco pares de *primers* que amplificaram éxons e a região promotora do gene da globina β. O produto da amplificação foi seqüenciado e os cromatogramas, analisados por programas de computador (Phred, Phrap e Consed).

**RESULTADOS:**

Foram encontradas duas mutações responsáveis pela doença;-101 (C > T) e códon 39 (C > T).

**CONCLUSÕES:**

Este caso representa a primeira descrição da mutação −101 (C > T) na população brasileira e está associado a evolução clínica benigna.

## INTRODUCTION

Thalassemias are the most common monogenic diseases and are characterized by hypochromic and microcytic anemia, which results from the absence (β^0^) or reduction (β^+^) of the synthesis of b-globin chains. The main pathological mechanism of this disorder is the excess of a chains that precipitate blood cell precursors, thereby inducing their premature destruction and making them incapable of effective erythropoiesis. Three main factors are responsible for the clinical manifestations of thalassemia intermedia: ineffective erythropoiesis, chronic anemia, and iron overload. The severity of these factors depends on the underlying molecular defects that determine the relative excess of a-globin chains. This is an extremely heterogeneous disorder and more than 180 mutations have been described around the world, causing different degrees of illness.^1^

In the homozygous state, thalassemias are generally classified clinically as either transfusion-dependent, denominated Cooley anemia or thalassemia major, or not requiring regular transfusions, presenting a benign clinical course and denominated thalassemia intermedia.

The aim of this study was to detect mutations in the β-globin genes of a 72-year-old Brazilian male patient of Italian descent with thalassemia intermedia.

## METHODS

DNA was extracted and purified by means of K proteinase, phenol and chloroform, as previously described.^2^ The DNA amplification method was followed, using 5 μl 10X Buffer (200 mM Tris-HCl, pH 8.4; 500 mM KCl; Gibco BRL Life Technologies); 5 μl 1 mM dinucleotide triphosphotide (dNTP); 3 μl 50 mM magnesium chloride (MgCl_2_); 0.5 μl, 0.5 units Taq polymerase; 1 μl oligonucleotide primer 20 mol/μl; and 5 μl patient DNA. Five pairs of primers were used (P1: TCC TAA GCC AGT G, P5: TCA TTC GTC TGT TTC CCA TTC, B1: TTT GCT TCT GAC ACA ACT GT, 109: CCC TTC CTA TGC ATG AAG TTA ACC AT, 58: AAT CCA GCT ACC ATT CTG C, P7: GAC CTC CCA CAT TCC CTT TTT, Prom3: GGT GTC TGT TTG AGG TTG, Prom5: CCA AAT AAG GAG AAG ATA TG, Exon3: CTG TGG GAG GAA GAT AAG AGG, Exon5: GTG AGT CTA TGG GAC CCT TG).

The cycle began with 94 °C for 2 minutes, followed by 30 cycles of 94 °C for 30 seconds, annealing temperature variations from 53 to 64 °C for 45 seconds, 72 °C for 1 minute and last extension reaction at 72 °C for 7 minutes. The PCR products were displayed via agarose gel electrophoresis 1% with ethidium bromide.

The fractions were purified using the Gibco Concert kit. The sequence reactions were performed using 3 μl of DNA, 4 μl of Big Dye, 6 μl of save money, 1.3 μl of primer 5 mol/μl, and 5.7 μl of water. The PCR cycle began with 96 °C for 90 seconds, 25 cycles of 96 °C for 12 seconds, 50 °C for 6 seconds and 60 °C for 4 minutes. The PCR products were sequenced using the ABI PRISM 3700. The chromatograms were analyzed via the software programs PHRED,^3^ PHRAP^4^ and CONSED.^5^

## PATIENT AND RESULTS

The patient was a 72-year-old male with a clinical course characteristic of thalassemia intermedia, who did not require regular transfusions. He did not present the classical features of severe clinical complications that are seen in thalassemia major. He presented mild anemia and had undergone splenectomy at the age of 30 years. The hematological data showed microcytic and hypochromic anemia (hemoglobin = 7.9 g/dl, mean cell volume = 76 fl, mean cell hemoglobin = 26 pg) and the hemoglobin electrophoresis indicated fetal hemoglobin = 14.2%, hemoglobin A_2_ = 6.2% and hemoglobin A = 79.6%. One mutation was identified at the position – 101 (C > T) in the b-globin gene promoter in one allele and another mutation in codon 39 (C > T) in the other allele.

## DISCUSSION

The promoter mutation was classified as extremely mild and an association was made with the heterozygosis of the silent carrier phenotype, which is characterized by little or no hypochromia and microcytosis of the red blood cells. This mutation was first described in Italians. The other mutation, resulting from a stop codon, is also common in the Mediterranean population and is the most frequently-found mutation in Brazil.

## CONCLUSION

This study provides the first description of the – 101 (C > T) mutation in Brazil and emphasizes the clinical variability of homozygosis for beta thalassemia in the Brazilian population.
